# In Vitro Human Haematopoietic Stem Cell Expansion and Differentiation

**DOI:** 10.3390/cells12060896

**Published:** 2023-03-14

**Authors:** Yavor K. Bozhilov, Ian Hsu, Elizabeth J. Brown, Adam C. Wilkinson

**Affiliations:** MRC Weatherall Institute of Molecular Medicine, University of Oxford, Oxford OX3 9DS, UK

**Keywords:** haematopoietic stem cells, haematopoiesis, erythrocyte, megakaryocyte, neutrophil, monocyte, B cell, T cell, self-renewal, differentiation, expansion, in vitro

## Abstract

The haematopoietic system plays an essential role in our health and survival. It is comprised of a range of mature blood and immune cell types, including oxygen-carrying erythrocytes, platelet-producing megakaryocytes and infection-fighting myeloid and lymphoid cells. Self-renewing multipotent haematopoietic stem cells (HSCs) and a range of intermediate haematopoietic progenitor cell types differentiate into these mature cell types to continuously support haematopoietic system homeostasis throughout life. This process of haematopoiesis is tightly regulated in vivo and primarily takes place in the bone marrow. Over the years, a range of in vitro culture systems have been developed, either to expand haematopoietic stem and progenitor cells or to differentiate them into the various haematopoietic lineages, based on the use of recombinant cytokines, co-culture systems and/or small molecules. These approaches provide important tractable models to study human haematopoiesis in vitro. Additionally, haematopoietic cell culture systems are being developed and clinical tested as a source of cell products for transplantation and transfusion medicine. This review discusses the in vitro culture protocols for human HSC expansion and differentiation, and summarises the key factors involved in these biological processes.

## 1. Introduction

The haematopoietic system plays essential roles in human health and survival. Erythrocytes oxygenate our tissues and platelets (from megakaryocytes) prevent bleeding, while myeloid cells (including neutrophils and monocytes) and lymphoid cells (including B and T cells) fight against infections. A continuous production of new blood cells is required to maintain haematopoietic system homeostasis, a process known as haematopoiesis. Self-renewing multipotent haematopoietic stem cells (HSCs) and a range of intermediate haematopoietic progenitor cell (HPC) types sustain haematopoiesis throughout life. In the adult, haematopoietic stem and progenitor cells (HSPCs) largely reside in the bone marrow (BM), in a specialised microenvironment or “niche” where HSC maintenance, self-renewal and differentiation are strictly regulated [1,2,3]. Adult HSPCs can also be mobilised into peripheral blood (mPB). During development, HSPCs are primarily found in the fetal liver, and can also be collected from umbilical cord blood (UCB). Throughout life, most HSPCs are marked by the surface marker CD34, which is often used for enrichment.

A large research effort has gone into identifying and characterising HSCs. HSCs are functionally defined by their ability to stably reconstitute the entire blood and immune systems following transplantation. HSCs can be prospectively isolated using fluorescence activated cell sorting (FACS) based on surface marker expression. In humans, HSCs are commonly immunophenotypically defined as CD34^+^CD38^−^CD49f^+^CD90^+^CD45RA^−^lineage^−^ cells [4]. However, immunophenotypic HSCs display heterogeneous self-renewal and differentiation potentials. Functional long-term HSCs (LT-HSCs) support life-long blood production and are commonly quiescent, which is thought to help protect them from detrimental stress and maintains their capacity for self-renewal [2]. In the classical model of haematopoiesis, LT-HSCs can give rise to short-term HSCs (ST-HSCs), which have a limited self-renewal potential and can differentiate into multipotent progenitors (MPPs). MPPs can differentiate into lineage-committed progenitors such as those that give rise to myeloid cells (common myeloid progenitors; CMPs) and to lymphoid cells (common lymphoid progenitors; CLPs). Lineage-committed progenitors have a limited self-renewal capacity and differentiation potential, and give rise to specific mature blood and immune cell types [5].

**Figure 1:**
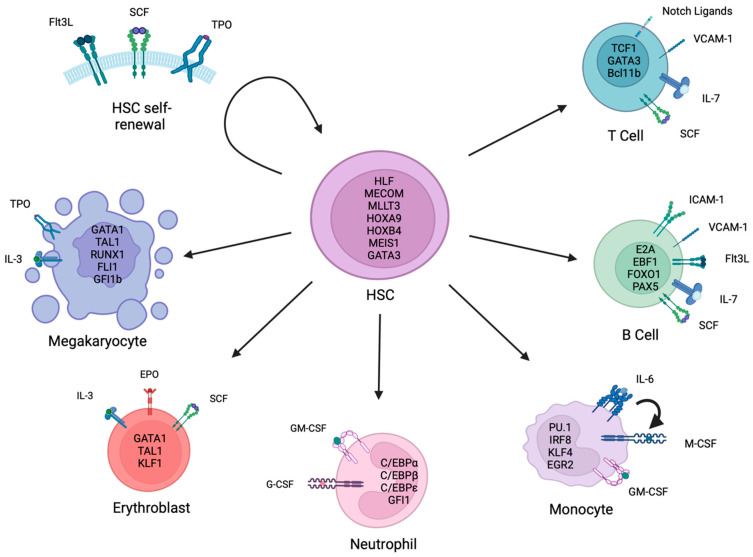
Key factors and pathways involved in haematopoietic stem cell self-renewal and differentiation. Haematopoietic stem cells (HSCs) can either self-renew to generate more HSCs or differentiate into the megakaryocyte, erythroid, myeloid (including neutrophil and monocyte) or lymphoid cell (including B cell and T cell) lineages. The key cytokine signalling pathways and transcription factors that regulate HSC self-renewal and differentiation are summarised. This figure was created using www.Biorender.com.

Over the years, the development of in vitro models of HSPC differentiation has been critical for deciphering the mechanisms regulating normal and disease haematopoiesis. Additionally, there is growing interest in the use of in vitro expanded and/or generated blood cells for clinical transfusion and HSC transplantation (HSCT) therapies. Here, we summarise the key methods and regulatory factors for in vitro human HSPC expansion and their differentiation into erythrocytes, megakaryocytes, myeloid cells and lymphoid cells (Figure 1).

## 2. In Vitro Maintenance and Expansion of HSPCs

**Figure 2:**
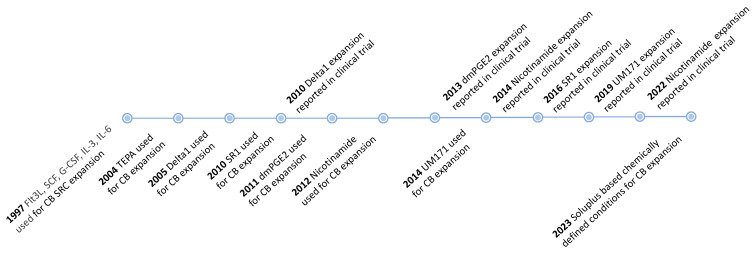
Timeline of culture conditions for improving HSC expansion. This figure was created using Microsoft PowerPoint.

A wide range of approaches have been tested in efforts to expand HSCs in vitro (Figure 2). HSC expansion protocols have powerful uses in basic research, but also have direct translational applications to help boost donor HSC numbers for HSCT therapies. In vitro HSC culture conditions are also necessary for ex vivo HSC gene therapies. However, stable HSC expansion in vitro remains a major challenge. The current gold standard assay for human HSC activity is the ability for serial engraftment in immunodeficient NOD-SCID or NOD.Cg-Prkdc^scid^ Il2rg^tm1Wjl^/SzJ (NSG) mice. These cells are referred to as NOD-SCID repopulating cells (SRCs) [6] and their absolute number is quantified by limiting dilution assays (LDAs) [7].

### 2.1. Signalling Pathways

Extracellular cues and intracellular factors regulate HSC cell-fate decisions and cell cycle entry and exit. Many conserved pathways form the intricate signalling network that regulates HSC cell fate to achieve the delicate balance between HSC self-renewal and differentiation that ensures life-long haematopoiesis. Downstream of the conserved signalling mechanisms sits a network of transcription factors (TFs) that regulate the expression of genes that participate in processes that maintain HSC self-renewal and potential. The complete list of HSC TFs is yet be elucidated, but a number of key regulators have been identified, including MECOM, MLLT3 and HOX genes (including HOXA9 and HOXB4), HLF, GATA3 and MEIS1 [8,9,10,11,12,13,14,15]. There are several signalling pathways that play an important role in regulating HSC cell-fate decisions, including Wnt and Notch signalling [16,17,18,19,20,21]. Other signalling pathways also play significant roles in HSC regulation, including the TGF-*β*/Smad, JAK/STAT and PI3K/AKT pathways [20,22]. These conserved signalling pathways are activated by a number of extrinsic factors, including small, secreted signalling proteins called cytokines.

Various cytokines have been used for in vitro maintenance and expansion of human HSPCs capable of repopulating immunodeficient mice, starting from the late 1990s [23,24]. The most widely used combination includes stem cell factor (SCF), thrombopoietin (THPO), Fms-like tyrosine kinase 3 ligand (FLT-3L) and interleukin-6 (IL-6) [25], which are discussed below. However, these basic expansion culture systems are incapable of stably maintaining HSC self-renewal, which leads to depletion of HSC activity and an expansion of HPCs in the cultures. As discussed below, combinations of these cytokines are also used in HSPC differentiation protocols. Other cytokines implicated in HSC maintenance include pleiotrophin (PTN), the angiopoietin-like proteins (Angptl) and sonic hedgehog (Shh). PTN is a heparin-binding growth factor secreted by cells in the BM niche, and it regulates HSC self-renewal via inhibition of protein tyrosine phosphatase-*ζ* (PTP*ζ*) [26,27,28,29]. The addition of PTN promotes expansion of human UCB HSCs [29]. The Angptl protein family plays roles in lipid metabolism, angiogenesis, inflammation, cancer cell motility and HSC expansion [30]. The addition of soluble Shh, a Hedgehog family protein, has been shown to induce human CD34^+^CD38^−^ cell proliferation and increase HSC repopulating ability by inhibiting bone morphogenetic protein (BMP) signalling [31]. While most HSPC expansion cultures are undertaken in traditional tissue culture plates, the novel use of 3-dimensional zwitterionic hydrogels has recently been shown to substantially improve cytokine-mediated HSC expansion [32].

*SCF:* SCF was first identified as the ligand for c-Kit (CD117) in mice [33] and cloned in 1990, where it was shown to promote the expansion of haematopoietic progenitor cells [34,35]. SCF was later shown to stimulate the survival and proliferation of HSCs [36,37]. SCF is expressed by cells in the BM niche to promote HSC maintenance, and it stimulates cell cycle entry by activating the PI3K/AKT/FOXO pathway [38,39,40]. Signalling downstream of SCF is reviewed extensively elsewhere [41].

*THPO:* THPO and its receptor MPL were discovered and cloned in 1994. THPO was first shown to promote the development and maturation of megakaryocytes [42,43,44,45,46,47]. However, THPO is also required for HSC maintenance and expansion after transplantation [48,49,50] through its ability to induce self-renewing cell division [51], and loss of THPO signalling causes a decrease in HSC numbers [52]. THPO signalling via the MPL receptor-ligand complex results in the activation of multiple signal transduction pathways, including Janus kinase/signal transducer and activator of transcription (JAK/STAT), mitogen-activated protein kinase/extracellular signal-regulated kinases (MAPK/ERK) and phosphoinositide 3-kinase/protein kinase B (PI3K/AKT) [53,54,55,56].

*FLT3L:* FLT3L was first cloned in 1993 and was found to stimulate the growth of HSPCs [57,58]. It also plays a key role in lymphoid differentiation, which is discussed in the lymphoid differentiation section below. FLT3L binds and activates the receptor tyrosine kinase (RTK) Fms-like tyrosine kinase 3 (FLT3) [59]. Activation of FLT3 by FLT3L activates the downstream PI3K, MAPK/ERK and JAK/STAT3/5 pathways, and also induces Src kinase activity [59]. Constitutive activation of FLT3L signalling drives proliferation of undifferentiated myeloid progenitors [60,61]. This is mirrored in vitro, where FLT3L promotes expansion of myeloid progenitors without differentiation to mature cells, in synergy with IL-3, GM-CSF, G-CSF and M-CSF [62,63]. Signalling downstream of FLT3 is reviewed in detail elsewhere [59].

*IL-6:* IL-6 is a pleiotropic cytokine which modulates expansion of multiple cell types, including HSPCs, B cells, T cells and monocytes [64]. IL-6 signalling is mediated by two different pathways: classic and trans-signalling. In classical signalling, IL-6 binds to a secreted form of the IL-6 receptor (IL-6R), whereas in *trans* signalling it binds membrane-bound IL-6R. In both cases, ligand bound IL-6R subsequently interacts with GP130, which facilitates recruitment of JAK1/JAK2/TYK2 and SRC kinases [65,66,67]. The activated JAK proteins phosphorylate tyrosine residues of GP130, which act as docking sites for proteins that initiate PI3K/AKT, RAS/RAF/MEF/MAPK and JAK/STAT1/3/5 signalling [64,65]. IL-6 signalling is reviewed in detail elsewhere [64,65].

*Notch ligands:* Notch signalling regulates various processes in HSPCs in a context-specific manner [19] and several Notch ligands have been shown to maintain HSC function. Jagged1, a Notch ligand, enables a modest HSC expansion in vitro and enhances long-term engraftment in immunodeficient NSG mice [68,69]. Additionally, expression of human Jagged1 in a transgenic hJ1-NOG mouse model induced a drastic increase in the expansion of transplanted human UCB HSCs [70]. Delta1, another Notch ligand, was used in combination with cytokines for in vitro expansion of human CD34^+^CD38^−^ UCB HSPCs. This showed an over 100-fold increase in HSPC numbers, higher SRC frequency and enhanced short-term repopulating activity in a low Delta1 dose culture [71,72,73]. A phase I clinical trial was performed using Delta1-expanded combined with non-expanded UCB HSCs and demonstrated that the expanded fraction exhibited rapid myeloid reconstitution, but only short-term repopulating activity [72].

*Co-culture systems*. Mimicking the BM niche has also been explored using co-culture expansions system that employ mesenchymal stromal cells (MSCs). In a phase I clinical trial (NCT00498316) UCB CD34^+^ cells were cultured on STRO-3^+^ MSCs supplemented with SCF, THPO, FLT3L and G-CSF [74]. A 14-day expansion increased CD34^+^ cell numbers by 30.1-fold and reduced time for haematopoietic recovery, but showed an impaired engraftment ability. More recently, a 3D MSC culture system composed of multicellular spheroids embedded in extracellular matrix has been shown to mimic the functionality of BM niche cells, maintain the expression of stem cell markers and release haematopoietic maintenance factors [75,76]. These approaches show promise as an in vitro tool to study the microenvironment in which HSCs reside.

### 2.2. Small-Molecules

Due to the limited success of in vitro HSC expansion using recombinant cytokines, the field has turned to implementing small-molecule approaches to improve in vitro HSC expansion, with several now tested in HSCT clinical trials. These small molecules are often used in combination with recombinant cytokines. This has yielded a large selection of new druggable pathways implicated in HSC maintenance and expansion, as summarised below and in Table 1.

*Tetraethylenepentamine (TEPA):* Expansion of UCB-derived CD34^+^ HSPCs with TEPA resulted in HSPC expansion and enhanced repopulating capacity in immunodeficient NSG mice [77,78]. TEPA is a high affinity copper chelator which was used to demonstrate that low copper conditions inhibited differentiation of UCB-derived HSPCs and promoted their proliferation [79,80]. The first phase I/II clinical trial to evaluate TEPA expanded UCB HSCs reported limited cell expansion and slow neutrophil and platelet recovery, but had high overall survival [81]. However, a second clinical trial reported higher cell expansion rates, faster haematopoietic recovery and had survival rates similar to the control group [82].

*StemRegenin-1 (SR1):* SR1 was identified from a small-molecule screen of a 100,000-compound library designed to detect compounds capable of promoting UCB-derived HSPC expansion. In combination with cytokines, the addition of SR1 led to a 50-fold expansion of UCB CD34^+^ HSPCs and a 17-fold increase in SRC numbers [6]. SR1 is a purine derivative which inhibits the aryl hydrocarbon receptor (AHR) in human HSPCs. Phase I/II clinical study with SR1-expanded UCB units has reported significant CD34^+^ HSPC expansion, enhanced engraftment and rapid haematopoietic recovery [83]. However, they observed a high incidence of transplant-related mortality and low overall survival. Two phase II trials (NCT03406962 and NCT03674411) are currently evaluating the use of SR1-expanded UCB cells for the treatment of haematologic malignancies and inherited metabolic disorders.

*Nicotinamide (NAM):* NAM was found to enhance UCB-derived CD34^+^ HSPC expansion 80-fold, and transplantation assays showed an increase in SRCs and improved engraftment potential by enhancing CXCR4-CXCL12-based homing. NAM is a form of vitamin B3 which selectively inhibits the histone deacetylase Sirtuin1 (SIRT1), and inhibition of SIRT1 using the selective inhibitor EX-527 was found to mimic NAMs effect on HSPC expansion [84]. Two phase I/II clinical studies with NAM-expanded UCB units have showed a low incidence of long-term engraftment failure after expansion and high survival rates [85,86]. A phase III trial (NCT02730299) on patients with haematologic malignancies is ongoing.

*Prostaglandin E2 (PGE2):* PGE2 is a potent inflammatory mediator that is generated by cyclooxygenase 2 (COX2) and it can regulate the stability of *β*-catenin via cAMP/PKA signalling to activate the Wnt pathway [87,88]. A stable PGE2 derivative, 16,16-dimethyl-PGE2 (dmPGE2), was shown to increase CFU activity and SRC frequency in mice [89]. Expansion of UCB with dmPGE2 improves HSC self-renewal and enhances engraftment in NSG mice [90]. A phase I clinical trial using dmPEG2 showed a rapid haematopoietic recovery and higher long-term engraftment from UCB units treated with dmPEG2 [91]. Modulating endogenous PGE2 levels is also an attractive method for improving HSCT efficacy. One compound, SW033291, was identified in a 230,000 small-molecule screen. SW033291 inhibits prostaglandin-degrading enzyme 15-PGDH and raises PGE2 levels in the BM [92]. SW209415, a second generation 15-PGDH inhibitor, was shown to enhance human HSC homing and engraftment in xenograft transplants [93].

*UM729 and UM171:* UM729 was identified from a small-molecule screen of a 5280-compound library designed to detect compounds capable of promoting expansion of human CD34^+^ HSPCs [94]. Structure-activity relationship (SAR) optimization of over 300 UM729 analogues yielded UM171 as the top candidate with more than 10-fold higher HSPC expansion activity. Both UM729 and UM171 are pyrimidoindole derivatives whose complete mechanism of action is not understood, but are thought to act to repress genes involved in erythroid and megakaryocytic differentiation through degradation of the epigenetic regulator lysine-specific histone demethylase 1A (LSD1) [94,95]. Transplantation assays showed that expansion with UM171 enhances LT-HSC frequency over 13-fold and maintains long-term repopulating capacity [94]. A phase I/II clinical study with UM171-expanded UCB units showed no graft failure after expansion and high survival rates [96]. Three more clinical trials (NCT03441958, NCT03913026 and NCT04103879) are under way to further evaluate the safety and efficacy of UM171 expanded HSCs in treating haematologic malignancies. The synthesis of novel pyrimidoindole analogues for HSC expansion remains an attractive approach and there are more pyrimidoindole compounds pending evaluation [97].

*THPO Mimetics:* Thrombopoietin (THPO) signalling through its receptor c-MPL plays an important role in HSC self-renewal. The small-molecule c-MPL-agonist NR101 induces activation of STAT5 and accumulation of HIF-1*α* to increase expansion of CD34^+^ HSPCs and total SRC numbers in culture [98]. Eltrombopag, a small molecule activator of c-MPL, can affect haematopoiesis in a THPO-independent pathway through its iron chelating activity. Eltrombopag reduces iron concentration in HSPCs, which in turn decreases cellular reactive oxygen species (ROS) levels and promotes human HSPC self-renewal in vitro [99,100].

*MAPK Inhibition:* MAPKs regulate a wide variety of cellular processes, including cell growth, migration, proliferation, differentiation and survival. The three major MAPKs–extracellular-signal-regulated kinases (ERKs), Jun amino-terminal kinases (JNKs), and stress-activated protein kinases (p38MAPKs) can be regulated by cytokines and growth factors that play crucial roles in haematopoiesis [101]. There have been a number of studies demonstrating that targeting MAPKs may be a promising strategy for promoting HSPC expansion. Inhibition of p38 by SB203580 promotes the expansion, self-renewal and repopulating capacities of UCB HSPCs [102]. SAR studies have yielded an analogue of SB203580 called C7, which showed enhanced HSC expansion activity and promoted long-term repopulation of UCB-derived HSCs in primary and secondary NSG mice recipients [103]. JNK inhibition by JNK-IN-8 can expand human UCB HSPCs, increase SRC frequency 3.88-fold in primary recipients and has demonstrated engraftment in secondary recipients [104,105].

*P18 Inhibition:* The cyclin-dependent-kinase (CDK) inhibitor p18 regulates the cell cycle by inhibition of the CDK4/6 signalling pathway. Absence of p18 promotes self-renewal and expansion of HSCs in vitro [106,107,108,109,110]. Potent p18 inhibitors P18IN011 and P18IN003 were identified from an in silico screen and could support in vitro expansion of mouse HSCs with improved engraftment capacity in serial transplants [108]. Another p18 inhibitor that was developed in silico, called 005A, can promote robust in vitro expansion of human UCB-derived HSPCs and enhance repopulating capacity [110].

*Histone Deacetylase (HDAC) Inhibition:* Epigenetic modifications have a major role in cell-fate decisions and the proteins that are involved in their establishment, maintenance and removal are an appealing target for pharmacological intervention. Valproic acid (VPA) is a non-specific HDAC inhibitor that has been commonly used for the treatment of a number of neurological disorders. VPA has been shown to promote HSPC proliferation and maintenance, and it upregulates HSC self-renewal genes such as *HOXB4* [111,112]. There is evidence to suggest that HSPC expansion induced by VPA is accompanied by cellular reprogramming [113,114,115]. VPA treatment promotes HSC multi-lineage engraftment in serial transplantation of expanded UCB-derived HSPCs in immunodeficient NSG mice [115,116].

*Histone Acetyltransferase (HAT) Inhibition:* Garcinol and isogarcinol are non-selective HAT inhibitors identified in a natural compound screen for HSC expansion activity [117]. Garcinol facilitated a 4.5-fold expansion of human UCB-derived HSC and increased the expression of a key HSC factor, *HLF*, which was accompanied with a doubling of SRC numbers in immunodeficient NSG mice.

*DNA Methyltransferase Inhibition:* Combined treatment with 5-aza-2′-deoxycytidine (5azaD), a DNA methyltransferase inhibitor, and trichostatin A (TSA), a HDAC inhibitor, enhances UCB HSPC expansion and engraftment potential in immunodeficient NSG mice [118]. There is evidence to suggest that the combination of 5azaD and TSA preserved HSC potential by reducing the rate of cell division, whilst promoting stem cell maintenance as genes involved in self-renewal, including *HOXB4*, were upregulated alongside cell cycle inhibitors *p21* and *p27* [119,120].

*BET inhibition:* CPI-203 was identified in a small HSPC expansion and engraftment screen for small-molecules targeting bromodomain-containing proteins (BCPs). BCPs take part in the regulation of gene expression by recognising specific epigenetic modifications. CPI-203 inhibits BCPs that harbour a specific bromodomain and extra-terminal motif (BET) domain. Short treatment with CPI-203 promotes UCB expansion and improves long-term repopulating capacity in vivo; however, it also promotes megakaryocyte expansion [121].

*Glycogen synthase kinase-3 (GSK-3) inhibition:* GSK-3 plays a major role in many signalling pathways critical for HSC fate determination such as Wnt, Notch and Hedgehog signalling. Treatment with GSK-3 inhibitor CHIR99021 enhanced haematopoietic recovery in immunodeficient NSG mice [122]. In combination with insulin, it inhibits differentiation and promotes self-renewal, leading to an increase in the number of engraftable HSCs [123]. UCB-derived HSPC expansion with another GSK-3 inhibitor, BIO, prolonged the cell cycle by upregulating the CDK inhibitor p57 and down-regulating cyclin D1. However, this resulted in an increased total number of HSCs and maintained the frequency of repopulating cells in vitro by promoting Notch and Angpt1/Tie2 signalling [124]. CHIR99021 has also been used in combination with the mTOR inhibitor rapamycin to promote HSC self-renewal and enhance engraftment in NSG mice [125].

Very recently, a serum albumin and cytokine-free HSC expansion system was described, which relies on the co-polymer Soluplus (or another polymer, polyvinyl alcohol) and solely employs small-molecules to stimulate HSC maintenance signalling. A PI3K activator (740 Y-P), a THPO-receptor agonist (Butyzamide) and the pyrimidoindole derivative UM171 were used to support 30-day expansion of UCB HSCs that are capable of serial engraftment in xenograft transplantation assays [126]. This culture system is a major step toward the development of chemically-defined HSC culture systems capable of long-term expansion coupled with stem cell maintenance.

**Table 1:** Summary of small molecules used to improve human HSPC expansion.

Compound	Activity	Expansion Effect on HSPC Culture	Refs.
TEPA	Copper chelator	30.5-fold CD34^+^CD38^−^ HSPCs 172-fold CFU activity Improved engraftment in xenograft transplants	[78]
		219-fold total nucleated cells 6-fold CD34^+^ HSPCs 37.8-fold CFU activity	[81]
		400-fold total nucleated cells 77-fold CD34^+^ HSPCs	[82]
SR1	AHR	50-fold CD34^+^ HSPCs 17-fold SRCs	[6]
		854-fold total nucleated cells 330-fold CD34^+^ HSPCs	[83]
NAM	SIRT1	80-fold CD34^+^ HSPCs 9-fold SRCs	[84]
		486-fold total nucleated cells 72-fold CD34^+^ HSPCs	[85]
		33-fold CD34^+^ HSPCs	[86]
dmPGE2	15-PGDH	1.4-fold CFU activity Improved engraftment in xenograft transplants 2.2-fold increase in BM homing	[90,91]
UM171	LSD1	13.4-fold SRCs	[94]
		35.4-fold CD34^+^ HSPCs Improved engraftment in xenograft transplants	[96]
NR101	THPO	4.9-fold CD34^+^CD38^−^ HSPCs 2.9-fold SRCs	[98]
Eltrombopag	THPO/Iron Chelator	1.42-fold CD34^+^CD38^−^ HSPCs	[99]
C7	p38	1554-fold CD34^+^CD38^−^CD45^+^CD45RA^−^ HSPCs 2.5-fold SRCs	[103]
JNK-IN-8	JNK	8-fold CD34^+^CD38^−^CD45RA^−^CD90^+^ HSPCs 3.88-fold SRCs	[104]
005A	p18	2.72-fold CFU activity Improved engraftment in xenograft transplants	[110]
VPA	HDAC	194.7-fold CD34+CD45+ HSPCs 6-fold SRCs	[116]
VPA	HDAC	213-fold CD34+ HSPCs 36-fold SRCs	[115]
Garcinol/ isogarcinol	HATs	4.5-/7.4-fold CD34^+^CD38^−^ HSPCs 2.5-fold SRCs (garcinol)	[117]
5azaD	DNMT	12.5-fold CD34^+^CD90^+^ HSPCs 9.6-fold SRCs	[118]
TSA	HDAC		
CPI-203	BET	5–10-fold Lin^−^CD34^+^CD38^−^CD45RA^−^CD90^+^CD49f^+^ HSPCs 1.5–3-fold SRCs	[121]
BIO	GSK-3	2-fold CFU activity	[124]
CHIR99021	GSK-3	7-fold total nucleated cells 5-fold SRCs	[125]
Rapamycin	mTOR		

## 3. In Vitro Differentiation to Megakaryocytes

Platelet-producing megakaryocytes are a rare blood cell type representing only 0.01% of all nucleated cells in the BM [127], and this makes them difficult to isolate and culture for research and for the production of platelets for clinical purposes. Platelets play major roles in haemostasis, thrombosis, inflammation, vessel constriction and repair, but only have a lifespan of up to 10 days, and need to be produced constantly by the body [128,129]. Failure to maintain platelet levels results in thrombocytopenia. Thrombocytopenia is commonly seen in haematological diseases, including haematopoietic aplastic anaemia, leukaemia and BM abnormalities, but may also arise from infectious, connective tissue and liver diseases, as well as chemotherapy/radiotherapy [128,130,131]. Platelet transfusion is an effective treatment that reduces the mortality caused by bleeding in these conditions. Platelets currently used in the clinic are solely donor-derived, and with the development of new clinical treatment options, the demand for platelet transfusion is increasing. This has led to an unmet need, and research has been focused on alternative strategies to obtain platelets, including in vitro platelet production.

Megakaryocytes are generated from HSCs through a stepwise process of differentiation [132,133]. A number of megakaryocyte differentiation protocols have been developed (Table 2), which commonly employ the platelet markers glycoprotein IIb/IIIa (CD41) and glycoprotein Ib (CD42b), and/or measure cell ploidy to assess culture purity. However, current protocols for producing megakaryocytes from HSCs suffer from low differentiation efficiency, inconsistent in vivo functional testing and difficulty in scaling up production due to high costs. One of the earliest attempts to create platelets in vitro from CD34^+^ PB HSPCs used human and aplastic canine serum to induce megakaryocyte differentiation with a very low differentiation efficiency and low platelet generation [134]. Later, differentiation efficiency was improved via the use of cytokines, namely THPO, either alone or in various combinations with SCF, IL-3 and IL-6. This demonstrated that the presence of THPO had the strongest effect on accelerating megakaryocyte differentiation [135]. Interestingly, elevated temperature has been shown to have a positive effect on differentiation of HSCs to megakaryocytes. Maintaining the culture at 39 °C promoted proliferation and megakaryocyte differentiation efficiency, and improved platelet output [136]. Large-scale differentiation processes using UCB-derived HSPCs and co-culture with telomerase-expressing human stromal cells and a three-phase cytokine cocktail system have produced larger numbers of platelets that were morphologically and functionally similar to those from plasma [137]. More recently, clinical-grade protocols for megakaryocyte generation from UCB-derived HSPCs have been developed using human albumin and a defined cocktail of cytokines and supplements [138]. Finally, 3D culture methods (roller-bottle cell culture system) have been able to further improve the efficiency of megakaryocyte and platelet generation from UCB-derived HSPCs [139,140].

Megakaryopoiesis is controlled by TFs which turn on the expression of megakaryocyte lineage-specific genes and suppress the transcriptional programmes of other lineages [141,142,143]. TFs found to be involved in this process include RUNX1, FLI1, GABPA, GATA2, LMO2, MYB, and NFE2 [144,145]. There is evidence that megakaryocyte and erythroid progenitors share a common bipotential precursor. In agreement with this, they have several TFs in common, including GATA1, FOG1, TAL1, and GFI1B [146,147]. Megakaryocyte development is coordinated by the temporal expression of these TFs and mediated at multiple levels by different cytokines, including THPO and interleukins.

*THPO:* The most critical megakaryocyte-stimulating cytokine is THPO. THPO regulates megakaryocyte differentiation from the earliest stages of megakaryopoiesis, and all progenitors primed to become megakaryocytes, including HSCs, express the THPO receptor MPL [148]. THPO is constitutively produced by the liver and sequestered by circulating platelets upon entry into the blood stream. When platelet count drops, for example, during haemorrhage, circulating levels of THPO increase and it enters the BM to stimulate megakaryopoiesis [149,150,151]. Loss of THPO signalling causes a drastic reduction in megakaryocytes and platelets [52]. However, patients with loss-of-function MPL mutations are still able to produce reduced numbers of platelets, suggesting that there is also a THPO-independent megakaryopoiesis pathway [152].

*Interleukins (IL):* The IL family comprises a disparate group of cytokines that are produced predominantly by leukocytes, and act as immunomodulators to elicit a variety of responses in cells and tissues throughout the body. They play a role in an array of processes, including cell proliferation, differentiation, migration and adhesion [153]. Interleukins that influence megakaryopoiesis include IL-1β and IL-6, which were found to upregulate THPO production resulting in increased platelet numbers in vivo [154,155], and IL-3, which was found to increase megakaryocyte proliferation in vitro without affecting maturation [156,157]. IL-1α has a THPO-independent function acting on mature megakaryocytes to promote shedding of proplatelets [158].

## 4. In Vitro Differentiation to Erythrocytes

Red blood cells (RBC), or erythrocytes, are the most abundant blood cell type, with ~2 × 10^6^ new erythrocytes produced per second in adults [159,160]. Reduced levels of healthy RBCs can lead to anaemia, defined by the inability of blood to adequately oxygenate the body’s tissues. Traditionally, the reduction of haemoglobin levels below 9–10 g/dL necessitates RBC transfusion [161,162]. RBC transfusion is conventionally employed in the treatment of haemorrhage, thalassaemia, chronic aplastic anaemia and chemotherapy/radiotherapy-induced anaemia, and is also used as supportive treatment for a range of genetic, autoimmune and neoplastic diseases. The demand for RBCs has continued to increase due the increasing burden of chronic disease and increasing severity of illness of intensive care patients brought about by an ageing population. The development of new in vitro methods for production could help to improve RBC supplies.

In vitro production of mature erythrocytes from HSCs utilises combinations of cytokines and/or stromal cells. It is commonly divided into several steps, which include erythroid lineage specification, erythroid progenitor expansion and erythroid maturation (Table 3). The purity of the cultures is commonly measured by the expression of the erythroid markers glycophorin A (CD235a), transferrin receptor (CD71) and/or Rhesus antigen (RhD), in combination with morphology analysis by May-Grünwald-Giemsa staining. One of the first protocols for erythroid differentiation of HSPCs utilised a 3-step process with specific cytokine combinations in serum-free culture medium to produce erythroid precursors that were capable of terminal maturation upon transplantation into immunodeficient mice [163]. In vitro terminal maturation and enucleation of erythroblasts was first achieved through the combined use of cytokines and co-culture on stromal cells [164]. Shortly after, stroma-free methods of producing erythroid cultures with incomplete but high percentages of mature/enucleated red blood cells were also developed [165]. As a proof-of-principal trial, a small number of RBCs generated from PB CD34^+^ HSPCs using stroma-free conditions were transfused into a health volunteer to monitor the lifespan of cultured RBC in humans (NCT0929266) [166]. However, the generation of large-scale, fully mature and clinically applicable RBCs has remained an obstacle to researchers for a long time, with the first clinical trial to transplant donor HSC-derived RBC launched only recently (ISRCTN42886452). The long-standing issues with incomplete enucleation of cultured erythrocytes that has delayed clinical applications has not prevented the use of cultured erythroid cells as a model to study erythropoiesis and related disorders, as the protocols available yield a highly proliferative and pure culture. Recently, large-scale (roller-bottle cell culture system) generation of functional enucleated RBCs from UCB-derived HSPCs has been demonstrated to be a safe source of RBCs in xenotransfusion studies [167].

The complex transcriptional programmes that define erythropoiesis are coordinated by a set of potent TFs [168,169], the expression of which is capable of transdifferentiating non-erythroid cells into erythroid cells [170]. GATA1 is the predominant key erythroid TF critical for erythroid lineage commitment, and differentiation and loss of GATA1 activity leads to impaired erythropoiesis [171,172,173,174,175,176,177]. Another key erythroid TF, KLF1, acts first to suppress the megakaryocyte programme and promote erythroid fate in the early stages of differentiation [178,179], and later triggers cell-cycle exit and chromatin condensation during terminal erythroid maturation, just before enucleation [180,181,182,183,184]. There are many more erythroid-associated TFs which form complex networks of coactivators or corepressors to modulate target gene expression. These include another major player, TAL1, which, along with LMO2 and LDB1, form a co-activator complex that binds GATA1 to activate the transcription of erythroid-associated genes [168,185]. Erythroid TFs are a downstream target of conserved signalling pathways which are activated by a number of extrinsic factors that control the differentiation, proliferation and survival of erythroid cells. The major erythropoietic cytokine is erythropoietin (EPO). However, other cytokines have been shown to promote erythroid proliferation and survival, including SCF [186,187] and IL-3 [188,189] (discussed above).

*Erythropoietin (EPO):* EPO is the most critical erythroid-stimulating cytokine. Human EPO was cloned in 1984 [190,191], and acts to promote the survival and proliferation of erythroid cells, starting with the earliest immature erythroid progenitors and persisting through to later stages of maturation. However, the EPO receptor (EPOR) is weakly expressed on erythroid cells, and its expression quickly decreases with terminal maturation [192,193,194,195]. EPO binding leads to EPOR dimerisation and activation of JAK/STAT to induce the erythroid transcriptional programme. Other signalling pathways stimulated by EPO include the MAPK and PI3K pathways, which act to promote survival and/or proliferation [196,197,198,199]. There is evidence to suggest that EPO may prime erythroid commitment in HSPCs, and that EPOR may be more broadly expressed in HPCs, indicating that erythroid lineage commitment may occur early during haematopoiesis [195,200,201,202]. EPO is produced in the liver and adult kidney, and acts to regulate the level of oxygen in the blood by modulating the number of circulating erythrocytes [203,204,205,206,207,208].

## 5. In Vitro Differentiation into Myeloid Cells

The myeloid compartment includes granulocytes (neutrophils, eosinophils and basophils), monocytes (macrophages and dendritic cells) and mast cells [209]. As the most numerous and extensively studied cells, we focus here on neutrophils and monocytes. Myeloid lineage specification is governed primarily by the C/EBP family and PU.1 TFs [210,211,212]. In mice, a high PU.1 concentration first regulates the decision between lymphoid and myeloid fates [213,214]. C/EBPα is then expressed in immature myeloid cells [215] and is critical for the CMP to GMP transition [216]. In myeloblasts, PU.1 and C/EBPα oppose each other’s expression, and a high C/EBPα/PU.1 ratio promotes granulopoiesis over monopoiesis [215,217,218]. C/EBPα then instructs neutrophil differentiation by activating the TF GFI1, whereas PU.1 activates IRF8, KLF4 and EGR2 to mediate monocytic differentiation [212,219,220,221,222]. Finally, C/EBPε is mainly expressed in late granulopoiesis and mediates neutrophil maturation [212,215].

### 5.1. Neutrophils

Neutrophils are the most abundant leukocyte in the blood, but have a lifespan of only 12–14 h, meaning that ~10^11^ neutrophils must be produced daily by the BM. Neutrophils play a key role in the innate control of infection by killing bacterial and fungal cells using phagocytosis and by releasing cytotoxic granules (degranulation) or nuclear material (neutrophil extracellular traps) [223,224]. Neutropenia is common following viral infection as a result of leukaemia and following chemotherapy or HSCT, and is associated with significant mortality due to the inability to control opportunistic infections [225,226]. Infusion of G-CSF or corticosteroid mobilised mature granulocytes has proven ineffective at reducing neutropenia, potentially due to insufficient cell dose or short intravascular residence time [227,228]. Infusions of non-HLA matched myeloid progenitors are undergoing clinical trials (NCT02282215, NCT01297543, NCT00891137, NCT01690520, NCT01175785, NCT01031368, NCT03399773, NCT04083170) as a treatment to reduce pathologies associated with neutropenia, such as the number of febrile days or microbiologically defined infections [227,229]. Thus, numerous protocols have been developed to expand neutrophils ex vivo, often with the aim of eventual clinical translation. Differentiation towards the granulocyte lineage is typically achieved with a combination of SCF, FLT3L, IL-3, GM-CSF and G-CSF [230,231,232,233,234,235] (see Table 4).

Early differentiation protocols often produced cells that were less than 50% immunophenotypic neutrophils [63,236], which are typically identified using a combination of Sialyl-Lewis X (CD15), carcinoembryonic antigen-related cell adhesion molecule 8 (CD66b) and/or FcγRIII (CD16). Subsequent protocols have aimed to improve purity. The use of protocols organised into stages first allows rapid expansion of myeloid progenitors with combinations of SCF, IL-3 or FLT3L, followed by a minimal set of lineage-defining cytokines, such as GM-CSF or G-CSF, which restricts differentiation towards the neutrophil lineage [231,232,233]. THPO or THPO mimetics can be included [63,237], but others report that THPO does not enhance growth of neutrophil progenitors [230,232] or can impair neutrophil differentiation [238]. Choi et al.[239] also noted that SCF and/or FLT3L minimally promote proliferation of myeloid precursors, but significantly increase the proportion of CD235a^+^ erythroid cells. Finally, switching from use of serum to serum albumin to supplement medium can also reduce multi-lineage differentiation [232]. Modern protocols are now capable of producing neutrophils demonstrating key functional characteristics such as chemotaxis, phagocytosis, oxidative burst, neutrophil extracellular trap formation and bactericidal activity at levels comparable PB neutrophils [232,233].

*IL-3 and GM-CSF:* IL-3 and GM-CSF promote the survival, differentiation and proliferation of a range of myeloid progenitor cells [236,240,241]. Indeed, the expression of IL-3 receptor (IL-3R, or CD123) is itself a marker for the common myeloid progenitor and granulocytic/monocytic progenitor cell identity [242]. Treatment of CD34^+^ HSPCs with IL-3 and/or GM-CSF induced proliferation and differentiation into cell types expressing early and late myeloid markers [236]. The IL-3R and GM-CSF receptor (GM-CSFR, or CSF2) are members of the type I cytokine receptor family, and share a common beta subunit [243,244,245]. Thus, engagement of either receptor triggers common downstream signal transduction pathways, including JAK2/STAT5, JNK-MAPK, PI3K and NFkB signalling. Signalling transduction downstream of IL-3R and GM-CSFR are extensively reviewed elsewhere [243,244].

*G-CSF:* G-CSF is a critical cytokine for neutrophil survival, proliferation and differentiation [228,234,246,247]. However, G-CSF only promotes high levels of granulocyte proliferation in synergy with SCF, FLT3L or IL-3 [232,236]. C/EBPε is induced only after addition of G-CSF to culture medium [248], and thus mature CD16^+^ neutrophils do not appear in liquid culture without the addition of G-CSF [63,248]. G-CSF influences the state of a myeloid progenitor by binding G-CSF receptor (G-CSFR, also known as CSF3R) and inducing its dimerization. G-CSFR lacks intrinsic kinase activity, but as a dimer it complexes with and activates protein tyrosine kinases, including JAK1/2, TYK2, SRC family kinases and SYK [249,250,251], which further activate MAPK/ERK and PI3K signalling [249,252,253]. JAK/STAT activation proceeds mainly via JAK1/2 and STAT1/3 [249,254], although STAT5 is also necessary for G-CSF induced differentiation in murine cells [255]. G-CSF mediated ERK1/2 activation is transient, and, paradoxically, ERK1/2 inhibition can direct differentiation towards the neutrophil rather than monocyte lineage [256,257]. Phosphorylation of C/EBPε and β by MAPK regulates expression of secondary granule genes and inhibits apoptosis [258], although the role of all G-CSFR dependent signalling is not yet fully understood. Signal transduction downstream of G-CSFR is reviewed elsewhere [251].

**Table 4:** Summary of protocols for in vitro neutrophil differentiation.

Cell Source	Method	Cells Generated	Reference
UCB CD34^+^	THPO, FLT3L, G-CSF	6–23% CD16^+^ (CD32^hi^CD64^hi^)	[63]
UCB or BM CD34^+^	IL-3, GM-CSF, G-CSF, M-CSF	100× expansion, 10–70% CD33^+^, <20% CD14/CD15^+^	[236]
PB CD34^+^ PBMCs	SCF, IL-3, GM-CSF, G-CSF	130–220× expansion, by morphology: 65% granulocytic, 11% band/segmented neutrophils, 5% monocyte/macrophages	[231]
UCB CD34^+^ HSPCs	SCF, IL-3, FLT3L, G-CSF	60–70% CD15^+^, 75% MPO^+^	[230]
G-CSF mobilised PB CD34^+^	SCF, FLT3L, G-CSF	30× expansion, 80% mature neutrophils (by morphology), CD16b^lo^,	[238]
BM CD34^+^	SCF, THPO, IL-3, G-CSF	76% band/segmented neutrophils, CD15^+^CD66^+^	[259]
UCB CD34^+^	SCF, G-CSF, THPO mimetic	5800× expansion, 61% metamyelocytes/band/segmented neutrophils, 73% CD15^+^	[237]
UCB CD34^+^	Phase I: SCF, IL-3, FLT3L, GM-CSF; phase II: G-CSF	8900–49,000× expansion, 59% CD66b^+^	[232]
UCB CD34^+^	SCF, FLT3L, IL3, THPO, EPO, with MS-5 stromal cells	Pan-myeloid differentiation, 23% CD34^+^, 12% CD14^+^ monocytes, 5% CD66b^+^ granulocytes, 8% CD41^+^ megakaryocytes, 11% CD235a^+^ erythrocytes	[240]
UCB or PB CD34^+^	Staged combinations of SCF, IL-3, FLT3L, GM-CSF, G-CSF	50–70× expansion, 70–92% CD15^+^, 43–57% CD66b^+^	[233]

### 5.2. Monocytes

Monocytes are a heterogenous cell population that play a key role in innate immunity, wound healing and chronic inflammation [260]. The most common source of monocytes (or subsequently in vitro differentiated macrophages) remains peripheral blood mononuclear cells (PBMCs), but this has limited their study or clinical use, as only a small proportion of PB monocytes are capable of proliferation in vitro [261]. Infusions of autologous monocytes/macrophages have been tested as a tumour treatment since the late 80s, but regardless of dose, schedule or cell source, only a partial response was seen in a few patients [227,262]. Testing of monocyte infusion continues for tumours such as ovarian carcinoma, where immune checkpoint inhibition shows limited efficacy (NCT02948426) [263,264]. Monocyte or macrophage infusions are also being developed as a treatment for inflammatory diseases such as liver cirrhosis (EudraCT number 2015-000963-15) [265,266] and ischaemic stroke (NCT024335090) [267].

In recent years, several methods for the in vitro differentiation of monocytes have been developed and may facilitate a greater understanding of monocyte biology. These typically use a combination of Siglec-3 (CD33), FcγRI (CD64), the lipopeptide receptor CD14 and/or CD16 as monocyte markers to assess culture purity. The cytokines IL-6 and M-CSF direct HSPCs towards the monocyte lineage, and IL-6 specifically favours macrophage rather than dendritic cell differentiation [268,269]. Early protocols using M-CSF and IL-6 induced differentiation of PB-derived CD34^+^ HSPCs into the monocyte lineage without expansion [270], but M-CSF or IL-6 alone (or in combination with other cytokines) do not promote proliferation [271]. Subsequent inclusion of early-acting cytokines such as SCF, IL-3, or FLT3L then supported monocyte expansion alongside differentiation (see Table 5). The addition of vitamin D3 can also bias the specific monocyte subsets produced [272], and the use of IL-2 can generate novel minor subsets (CD56^+^ CD33^+^) of monocytes [273]. Macrophages produced from the most recent protocols have gene expression profiles similar to PB monocytes and demonstrate key phenotypes including phagocytosis, adhesion and osteoclast generation [271,274].

Monocytes can be further differentiated into dendritic cells or polarised towards different sets of macrophages, but this will not be discussed in depth here. Briefly, M1 pro-inflammatory macrophages can be generated with lipopolysaccharide (LPS), IFN-γ or GM-CSF [234,275,276], whereas various M2 subtypes can be generated using combinations of IL-4, IL-10, IL-13, glucocorticoids, IL-1β or LPS [234,275]. The dendritic cell lineage is typically established using GM-CSF and IL-4 [241,244,277,278].

*M-CSF*: M-CSF is a potent factor driving the survival and differentiation of monocytes and macrophages [279,280,281]. Enforced expression of the M-CSF receptor, M-CSFR (also known as CSF-1R, or CD115), in UCB-derived CD34^+^ or even B cells promotes monocyte differentiation in vitro and in vivo [282,283]. M-CSFR is a class III receptor tyrosine kinase [251], which, upon activation, complexes with and phosphorylates GRB2/SOS, the p85 subunit of PI3K, SFK and CBL [284,285,286,287]. Activation of the PI3K/AKT pathway promotes survival, motility and proliferation in macrophages [286,288,289,290]. In mouse models, M-CSF stimulates more potent and sustained activation of Erk1/2, which is necessary for monocyte development and proliferation [257,289,290,291,292]. M-CSF dependent Src kinase activity instructs the monocyte fate by inducing expression of PU.1 [280], although the specific roles of the other signalling pathways are not yet completely described. Downstream signalling is reviewed elsewhere [287].

**Table 5:** Summary of protocols for in vitro monocyte differentiation.

Cell Source	Method	Cells Generated	Reference
G-CSF mobilised PB CD34^+^	M-CSF, mast cell growth factor (MGF), IL-6	No expansion, 55% CD33^+^CD14^+^, 62% CD33^+^HLA-DR^+^	[270]
G-CSF mobilised PB CD34^+^	SCF and IL-2	Majority CD33+, 2.5% CD33^+^CD56^dim^, NK-like monocytes	[273]
UCB CD34^+^	SCF, IL-3, FLT3L and M-CSF	300× expansion, 45% CD14^+^, 22% CD16^+^	[272]
G-CSF mobilised PB CD34^+^	SCF, FLT3L, IL-3, IL-6, GM-CSF, M-CSF with 0.5% buminate	360× expansion, 90% CD11b^+^, 65% CD64^+^, 45% CD14^+^	[278]
G-CSF mobilised PB	SCF, FLT3L, IL-3, IL-6 M-CSF with 1% Buminate	90% lysozyme positive, CD14^+^CD64^+^CD16^+^HLA-DR^+^	[271]
UCB CD34^+^	SCF, IL-6, FLT3L or SCF, THPO and FLT3L and viral transduction of M-CSF	350× expansion, 70–80% CD33^+^CD14^+^	[283]

## 6. In Vitro Differentiation into Lymphoid Cells

In the classical model for lymphoid differentiation, HSCs differentiate into LMPPs and then into CLPs, which then commit to B, T or natural killer (NK) cell lineages. Here, we will focus on the differentiation into B and T cells, given the larger numbers of studies into these lineages.

### 6.1. B Cells

Mature B cells are characterized by surface expression of mature immunoglobulin (Ig), produced from successfully recombined heavy and light chain loci. Post-natal B cell lymphopoiesis occurs in the BM in vivo. In humans, several B cell precursors have been identified. CLPs (CD34^+^CD38^+^CD45RA^+^CD10^+^) differentiate into pro-B cells (CD34^+^CD10^+^CD19^+^IgM^−^), then pre-B cells (CD34^−^CD10^+^CD19^+^IgM^+^), then immature B cells (CD34^−^CD19^+^IgM^+^) and finally mature B cells [293,294]. Pro-B cells are the earliest committed B cell population, and the first to undergo Ig heavy chain rearrangement. It has become well-established that the entry into B cell fate depends on the activation of four key TFs (E2A, EBF1, FOXO1 and PAX5 [295]), which act on each other in a stepwise fashion. In CLPs, the E2A gene product E47 is essential in normal lymphocyte development as well as in B lymphocyte V(D)J recombination [296]. E2A, in turn, acts on EBF1 and FOXO1; EBF1 acts on FOXO1; and E2A, EBF1 and FOXO1 all act on PAX5 [295]. PAX5, in turn, has been found to be a master regulator TF which simultaneously represses inappropriate B cell lineage genes and activates B cell lineage-specific genes [297]. Several culture systems have been developed to support in vitro B cell development (see Table 6), but generally require integrin signalling and IL-7.

*Integrins:* Leukocyte integrins have long been known to play essential roles in localization, activation and differentiation of human lymphocytes, and the first paper to identify an essential component of B cell differentiation focused on BM stromal contact. In 1990, it was shown that murine pre-B cells adhere to fibronectin, but lose this ability as they mature [298]. Notably, however, it was found soon after that human B cell precursors require VLA-4/VCAM-1 interaction (not the VLA-4/fibronectin interaction) for long-term development. While the exact mechanism remains unclear, subsequent studies have shown that cross-linking of VLA-4 with VCAM-1 leads to induction of tyrosine kinase pathways in B cells [299,300]. Notably, a more recent protocol for feeder-free B cell differentiation used immobilized ICAM1-Fc-coated plates. However, this publication attributed the use of ICAM-1 (which binds LFA-1 expressed on CD34^+^ HSPCs and B cells) to promote development of lymphoid progenitors, and found that differentiation into B cells was independent of ICAM-1 [301], demonstrating that differentiation of CD34^+^ HSPCs into B cells is possible even without contact elements.

*IL-7:* Following the discovery that B cells could be cultured with stromal cells, the first stromal cell growth factor isolated was IL-7 (found by screening a cDNA library prepared from a stromal cell line [302]), and IL-7 quickly made its way into a staple stromal culture supplement [303]. Thereafter, it was found that IL-7 promotes B cell clonal proliferation (but not differentiation) [304], and it was found that IL-7 specifically promoted proliferation of pro-B cells but not pre-B cells [305]. Closer investigation into the IL-7 receptor has revealed that it possesses a number of functional intracellular domains, including SRC family-related regions, JAK/STAT related regions and PI3K/AKT activation regions [306], indicating a wide range of downstream functions related to proliferation. CLPs from Il-7-knockout mice have been shown to have impaired B cell generation ability, likely due to the observed lower expression of key B cell TFs EBF and PAX5 [307]. However, ectopic overexpression of EBF restores B cell development [307,308], indicating EBF to be a primary downstream target of IL-7. Further investigations have revealed induction of EBF1 through the JAK/STAT5 signalling pathway [308]. Notably, FLT3L has also been found to act in synergy with IL-7, albeit it through independent signalling pathways [309].

*Other factors:* In addition to SCF, FLT3L and IL6 (all introduced above), additional cytokines including IL-4 [310] have been found to promote B cell lymphopoiesis. However, it is likely that they exert their influence primarily in latter phases of B cell development, as is the case for IL-2, IL-5 and IL-10 [311,312,313]. Additionally, it has been found that Activin A and TGF-β1 are negative regulators for early B cell lymphopoiesis, and antibodies and inhibitors targeting these factors can increase B cell lymphopoiesis [314]. Notably, as many in vitro cultures are successful in generating B cell precursors even in the absence of these cytokines and factors, it is likely that these cytokines are not necessary for B cell lineage commitment.

**Table 6:** Summary of protocols for in vitro B cell differentiation.

Cell Source	Method	Cytokines	Cell Generated	References
Nucleated BM cells	BM fibroblast feeder layer	None	CD10^+^/CD20^+^ B-lineage cells	[315]
UCB CD34^+^	Murine fetal stromal feeder cell layer (MS-5) with cytokines	SCF, G-CSF	CD19^+^/IgM^+^	[316]
BM CD34^+^Lineage^−^CD38^−^	Murine fetal stromal feeder cell layer (AFT024) with cytokines	IL-2, SCF, FLT3L, IL-7, IL-3	CD10^+^/CD19^+^ B-lineage cells	[317]
Fetal BM CD34^+^Lineage^−^	Human fetal BM stromal cells	None	IgM^+^ immature B cells	[318]
UCB CD34^+^	Human BM stromal cell layer with cytokines (and additional antibodies)	SCF, FLT3L; (anti-Activin A and anti-TGFβ antibodies)	IgM^+^ immature B cells	[314]
UCB CD34^+^	Murine stromal cell layer (S17)	Il-10, IL-4, FLT3L, IL-2	IgM/IgG-secreting B cells	[319,320]
Fetal liver CD34^+^	Murine stromal cell (OP9) with cytokines	TSLP	Mature CD34^−^CD38^+^CD19^+^IgM^+^IgD^+^ B cells	[321]
UCB or BM CD34^+^	ICAM-1 coated plate with cytokines	IL-6, SCF, FLT3L, IL-7	IgM^+^ CD19^+^ immature B cells	[301]
UCB CD34^+^	Cytokines only	IL-6, FLT3L, SCF, IL-7	CD10^+^CD79α^+^CD19^+^ pro-B cells	[322]

### 6.2. T Cells

The development of T cells in humans occurs primarily in the thymus and requires carefully mediated migration of T cell progenitors into thymic regions, which provide a specialised microenvironment for T cell differentiation. The earliest T cell precursors in the thymus are known as early thymic progenitors (ETPs) or double negative 1 (DN1) cells, defined as CD34^+^CD7^−^CD5^−^CD1a^−^ cells. As ETPs differentiate, they generate DN2 cells, which are committed to the T cell lineage. Notably, RAG-mediated rearrangement of the T cell receptor (TCR) is detectable in the DN2 phase, but presents predominantly in the DN3 (CD7^+^CD5^+^CD45RA^+^) phase, during which only cells with a productively rearranged TCR-beta and successfully formed pre-TCR complex are permitted to continue differentiation along the alpha/beta lineage. Subsequently, these cells become DN4 (or pre-DP cells), then immature single positive (ISP) cells (named for brief CD4 expression), then CD4^+^CD8^+^ double positive (DP) cells, which undergo TCR-alpha rearrangement and result in a completely assembled TCR. DP cells then undergo positive selection for MHC binding, then negative selection for autoreactivity, before finally becoming mature CD4^+^ or CD8^+^ single positive (SP) T cells.

One of the most critical signals is activation of the TF TCF1 (encoded by *TCF7*), which then results in subsequent expression of many T cell-specific TFs such as GATA3 and BCL11B [323]. Expression of GATA3 represses FLT3 expression, causing loss of B cell potential; similarly, activation of BCL11B (regulated in part by GATA3) prevents NK cell differentiation, and myeloid/DC potential is lost by PU.1 silencing. Activation of these three TFs (TCF1, GATA3, and BCL11B) allows progression from ETP stage through to DN2, upon which a host of other TFs (such as RUNX1/CBFB, GFI1, E2A, MYB and IKAROS) solidify the commitment to the T cell lineage [324]. 

T cells have important applications in cancer treatment, particularly as tumour-infiltrating lymphocytes (TILs) and chimeric antigen receptor (CAR) T cells. CAR T cell therapies require the production of large number of T cells; however, current methods of T cell production suffer from several problems. These include (1) peripheral blood T cells sourced for treatment often exhibit compromised (e.g., anergic or exhausted) function [325], and (2) the need for T cells to be sourced autologously prevents production of T cell therapies at scale. Protocols allowing for the in vitro differentiation of T cell precursors from HSCs could solve these problems [326]. Several culture systems have been developed to support in vitro T cell development (see Table 7), a process that is highly dependent on Notch signalling.

*Notch signalling:* One of the earliest discovered requirements for T cell differentiation was Notch signalling. Upon migration to the thymus, the thymic microenvironment provides the critical Notch ligand DLL4, which triggers proteolytic release of intracellular NOTCH1. Recent genome-wide studies have indicated that Notch signalling is related to many targets, but prominent targets include MYC, DTX1 and members of the HES and HRT family, as well as crosstalk with other signalling pathways including NFkB and hypoxia [327,328,329,330,331]. In particular, Notch signalling induced TCF1 expression, which, in turn, drives expression of key T cell TFs such as GATA3 and BCL11B [323]. As discussed below, various approaches have been used to stimulate Notch signalling within in vitro T cell differentiation culture systems.

*Stromal co-culture-based T cell differentiation:* The earliest T cell cultures used OP9 murine BM stromal cells retrovirally transduced with Notch ligand Delta-like-1 (DLL1) to create the OP9-DLL1 cell line. Murine fetal liver haematopoietic progenitor cells cultured on OP9-DLL1 were shown to support robust differentiation to DP T cells and some generation of mature SP T cells [332]. The culture system has since been successfully expanded to human UCB-derived HSPCs [333], and pro-T cells derived from these cultures can engraft in immune-deficient mice [334]. Notably, however, the T cells differentiated on OP9-DLL1 cells demonstrate high bias towards the CD8^+^ T cell lineage [335]. A second DLL1 expressing stromal cell type, the TSt-4 stromal line, is also used to support T cell differentiation from UCB-derived HSPCs [336]. Notably, while the OP9-DLL1 system remains widely used, subsequent studies have found DLL4 (and not DLL1) to be the critical Notch ligand, as DLL4 allows more efficient T cell differentiation [337], and DLL1 deletion (but not DLL4 deletion) [338] from thymic epithelial cells has no effect on T cell lymphopoiesis.

*Artificial Thymic Organoid (ATO)-based T cell differentiation:* ATO cultures are characterized by a 3D aggregate of mixed HSPCs and thymic cells, cultured on top of a porous membrane and placed into liquid media [339,340,341,342]. As with stromal cell monolayers [335], ATO differentiation has been demonstrated to generate mature naïve SP T cells [342]. However, the efficiency of ATO differentiation into mature T cells has been found to outperform stromal cell monolayers [342]. While the reason behind the improved efficiency remains unknown, it is possible that the 3-dimensional nature of the ATO increases the heterogeneity and likelihood of thymic-like niches, which may facilitate movement of T cells towards regions within the ATO which promote T cell maturation. Furthermore, the increased density between T cells and stromal cells in ATOs may also promote crosstalk, which enhances T cell maturation.

*Stromal-free T cell differentiation:* Due to inherent variability introduced into cell culture from the use of feeder cells, several attempts have been made to differentiate HSCs into T cells using feeder-free culture. The essential component of these feeder-free cultures has been the use of Notch ligands fused to the antibody Fc region, and subsequent immobilization onto a plate [343,344]. Immobilized DLL4 cultures have been validated for UCB and mPB HSPC differentiation [72,345,346]. However, these studies also highlighted differences between immobilized DLL4 cultures and in vivo thymic development, namely that development seemed blocked at the pre-T cell phase. Nevertheless, transplanted DLL4-cultured T cells still successfully engraft in vivo [345]. Additional work on optimizing feeder-free cultures has identified a range of molecules which appear to boost T cell development, such as WNT3A [347], ascorbic acid [348] and VCAM-1 [349]. More recently, use of DLL4-microbeads instead of immobilized DLL4 has allowed conversion from a plate-based system to a bioreactor system, allowing production of clinically relevant numbers of T cells [350]. Notably, while the system demonstrated limited progression to the SP T cell phase, successful thymic engraftment into immunodeficient mice was observed, which paves the way for clinical translation.

*Other factors:* Other factors found to have an influence on T cell lymphopoiesis include IL-7, SCF, IL-3, IL-15, CXCL12 and TNFa. While IL-3 alone appears to stimulate myeloid-biased differentiation, when paired with TNFa there appears to be a strong proliferative effect towards lymphoid-biased differentiation [351]. CXCL12 (which binds CXCR4) has been found to be expressed on TECs [352], and promotes Notch-dependent differentiation [353]. Notably, a number of additional cytokines (such as IL-2 and IL-15) have also been implicated in proliferation of mature T cells [354,355].

**Table 7:** Summary of protocols for in vitro T cell differentiation.

Cell Source	Method	Cytokines	Cell Generated	Reference
UCB CD34^+^CD38^−^	OP9-DLL1 stromal feeder cell with cytokines	FLT3L, IL-7	CD4^+^CD8^+^ DP T cells	[333]
UCB CD34^+^CD38^−^CD3^−^CD19^−^	Culture on MS5 then transfer to DLL4-coated plate	IL-2, IL-15, SCF	CD7^+^CD3^+^	[356]
UCB CD34^+^CD38^−^	Murine stromal feeder layer (Tst-4/hDLL1)	None	CD5^+^CD7^+^ immature T cells	[336]
UCB CD34^+^	DLL4-coated plate with cytokines	SCF, THPO, FLT3L, IL-7	CD5^+^CD7^+^CD1a^+^ immature T cells	[345]
UCB CD34^+^	DLL4- and VCAM-1 coated plate with cytokines	SCF, THPO, FLT3L, IL-7	CD7^+^ pro-T cells	[349]
UCB CD34^+^	DLL4- and VCAM-1 coated plate with cytokines	SCF, THPO, FLT3L, IL-7, IL-3, TNFa	CD4^+^CD8^+^ DP T cells	[351]
UCB and BM CD34^+^	ATO system with cytokines	FLT3L, IL-7	CD4^+^ and CD8^+^ mature SP T cells	[342]
UCB CD34^+^	DLL4-coated microbeads with cytokines	FLT3L, SCF, IL-7	CD7^+^CD5^+^ immature T cells	[350]

## 7. Summary

A large research effort over the last five decades has led to a range of in vitro human HSPC maintenance, expansion and differentiation protocols. These methods provide useful models to study human haematopoiesis and generate cell products for cell therapies. However, current technical challenges in stably expanding pure populations of bone fide human HSCs in vitro limit the further development of these technologies to comprehensively study human haematopoiesis in a dish. By contrast, the establishment of stable in vitro culture conditions for the expansion and differentiation of embryonic stem cells has enabled major insights into human development. Further improvements in expansion and differentiation culture conditions for human HSCs should extend the utility of these systems.

## Figures and Tables

**Table 2:** Summary of protocols for in vitro megakaryocyte differentiation.

Cell Source	Method	Cells Generated	Reference
PB CD34^+^	human/canine serum	>95% CD41^+^ megakaryocytes	[134]
PB CD34^+^	THPO	>79% CD41^+^ megakaryocytes	[135]
UCB CD34^+^	THPO, SCF, IL-6, FLT3L	>80% CD41^+^, >50% CD41^+^CD42b^+^ megakaryocytes	[136]
UCB CD34^+^	Phase I: THPO, SCF, FLT3L, human stromal cells; Phase II: THPO, SCF, FLT3L, IL-11, human stromal cells; Phase III: THPO, SCF, FLT3L, IL-11	>0.5% CD41^+^ megakaryocytes, 4.2 × 10^5^ platelets/starting CD34^+^ cell	[137]
UCB CD34^+^	Phase I: THPO, SCF, IL-3; Phase II: THPO, IL-11	>85% CD41^+^CD42b^+^ megakaryocytes, 1.9 × 10^4^ platelets/starting CD34^+^ cell	[139]
UCB CD34^+^	Phase I: THPO, SCF, FLT3L, IL-3, SR1; Phase II: THPO, SCF, IL-3, IL-6, IL-11, GM-CSF	>70% CD41^+^CD42b^+^ megakaryocytes	[138]
UCB CD34^+^	Phase I: THPO, SCF, FLT3L, IL-6, SR1, C433, VPA; Phase II: THPO, SCF, IL-3, IL-6, IL-11, GM-CSF	>70% CD41^+^CD42b^+^ megakaryocytes	[140]

**Table 3:** Summary of protocols for in vitro erythroid differentiation.

Cell Source	Method	Cells Generated	Reference
UCB CD34^+^	Phase I: SCF, THPO, FLT3L, hydrocortisone; Phase II: SCF, EPO, IGF-I, hydrocortisone; Phase III: EPO, IGF1, hydrocortisone	>80% CD71^+^CD235a^+^ erythrocytes	[163]
UCB and PB CD34^+^	Phase I: SCF, IL3, EPO, hydrocortisone; Phase II: EPO in stromal cell co-culture; Phase III: stromal cell co-culture	>80% CD71^+^ erythrocytes	[164]
UCB CD34^+^	Phase I: SCF, EPO, IL-3 or SCF, EPO, IL-3, VEGF, IGF-II; Phase II: SCF, EPO; Phase III: SCF, EPO(reduced); Phase IV: plasmanate and mifepristone	>90% CD235a^+^RhD^+^ erythrocytes	[165]
PB CD34^+^	Phase I: SCF, IL3, EPO, hydrocortisone; Phase II: SCF, EPO; Phase III: EPO	>85% CD71^+^CD235a^+^ erythrocytes	[166]
UCB CD34^+^	Phase I: SCF, THPO, FLT3L; Phase II: SCF, FLT3L, EPO, IL3, GM-CSF; Phase III: SCF, FLT3L, EPO, IL3; Phase IV: SCF, EPO	>90% CD235a^+^ erythrocytes	[167]
